# Implicit attitudes to sexual partner concurrency vary by sexual orientation but not by gender—A cross sectional study of Belgian students

**DOI:** 10.1371/journal.pone.0196821

**Published:** 2018-05-08

**Authors:** Chris R. Kenyon, Kenny Wolfs, Kara Osbak, Jacques van Lankveld, Guido Van Hal

**Affiliations:** 1 HIV/STI Unit, Institute of Tropical Medicine, Antwerp, Belgium; 2 Division of Infectious Diseases and HIV Medicine, University of Cape Town, Cape Town, South Africa; 3 Faculty of Psychology, Open University of the Netherlands, Heerlen, The Netherlands; 4 University of Antwerp, Medical Sociology and Health Policy, Antwerp, Belgium; University of New South Wales, AUSTRALIA

## Abstract

High rates of sexual partner concurrency have been shown to facilitate the spread of various sexually transmitted infections. Assessments of explicit attitudes to concurrency have however found little difference between populations. Implicit attitudes to concurrency may vary between populations and play a role in generating differences in the prevalence of concurrency. We developed a concurrency implicit associations test (C-IAT) to assess if implicit attitudes towards concurrency may vary between individuals and populations and what the correlates of these variations are. A sample of 869 Belgian students (mean age 23, SD 5.1) completed an online version of the C-IAT together with a questionnaire concerning sexual behavior and explicit attitudes to concurrency. The study participants C-IATs demonstrated a strong preference for monogamy (-0.78, SD = 0.41). 93.2% of participants had a pro-monogamy C-IAT. There was no difference in this implicit preference for monogamy between heterosexual men and women. Men who have sex with men and women who have sex with women were more likely to exhibit implicit but not explicit preferences for concurrency compared to heterosexual men and women. Correlates of the C-IAT varied between men and women.

## Introduction

Sexual partner concurrency refers to the practice of having two or more partners in an overlapping time interval [1]. By increasing sexual network connectivity the observed large variations in the prevalence of this practice are thought to play a role in determining the large variations in the prevalence of STIs around the world [1–5]. Higher rates of concurrency have been reported in men having sex with men (MSM) compared with heterosexual men [6], in men compared to women [7, 8] and in certain ethnic groups in countries such as Kenya, South Africa, the United Kingdom and the United States [9–12]. There is ongoing debate about what determines the differences in concurrency prevalence. Studies have argued that a range of factors are responsible, including high migration rates [13–15], demographic factors such as gender imbalance [7], socio economic inequality [13, 16], gender-related sexual attitudes including the sexual double standard [17–20], social network-influences [21, 22], drugs and alcohol [23], perceived recent partner non-monogamy [24, 25] and a culturally sanctioned tolerance of concurrency [26–33].

One problem with much of this research is that it has relied on the self-reported prevalence of- and attitudes to concurrency. This is susceptible to a number of biases. First, assessments of partners concurrency have been shown to fairly inaccurate in certain studies which would lead to a misclassification bias [34, 35]. Second, deficiencies in respondents’ memory of the timing of past sexual partnerships may lead to inaccuracies in the prevalence of reported lifetime concurrency [35, 36]. Finally, widespread mononormativism (the presupposition that monogamy or serial monogamy is the most ethical form of relationship) and other factors have resulted in a social desirability bias that may lead to inaccuracies in assessing who has engaged in concurrency [37, 38]. A similar process may lead people to underreport the extent to which they explicitly endorse forms of concurrent partnering. This under-reporting could occur for two reasons. Firstly, people might not want to report pro-concurrency attitudes because they do not want others to know about them [39]. Secondly, people might not be able to report on their personal pro-concurrency attitudes because they do not even realize that they hold such an attitude [39].

Testing implicit attitudes towards concurrency may allow researchers to avoid these biases. Testing of implicit cognitions via the Implicit Association Test (IAT) has been shown to be particularly useful for socially sensitive topics [40–44]. Measures of implicit cognition assess cognitive processes that are unavailable to introspection and are thus less affected by self-presentational concerns. In socially sensitive domains, IAT and self-reports are typically only weakly correlated [40]. Several studies have found implicit measures to be better predictors of behavior than explicit measures in these sensitive domains [40, 43–45]. We are currently unaware of any studies that have assessed implicit attitudes towards sexual concurrency. This provided the motivation for developing a Concurrency-IAT (C-IAT) that measures the implicit associations that individuals hold towards concurrency in relation to monogamy. A better understanding of the social, demographic and explicit attitudinal correlates of favorable implicit attitudes to concurrency and how these relationships vary between groups may provide more insight into the determinants of concurrency. In this paper, we describe the C-IAT and its application in the testing of two hypotheses in a population of Belgian students. University students were chosen as this age group has been shown to have a higher prevalence of reported concurrency than older age groups [46, 47]. We hypothesized that there may be differences in implicit attitudes to concurrency between men and women and between groups by sexual orientation.

## Methods

### Concurrency-IAT description

Implicit Association Tests (IATs) are reaction-time measures that tap implicit associations without requiring conscious introspection [48]. Our C-IAT was conducted in Dutch and constructed using the attribute categories “positive/negative” and the target categories “monogamy/multiple partners.” Participants had to categorize words, using the ‘z’ and ‘m’ keyboard keys, as either positive or negative (4 word stimuli each) and pictures as either depicting two people in a monogamous relationship or two people of which one had another partner (4 pictures in each category). The stimuli used in the IAT as well as links to the actual IAT used are provided in S1 IAT Test.

Our C-IAT consisted of five different blocks. In the first block (16 trials), participants were asked to categorize words appearing in the center of the computer screen according to two attribute categories (positive/negative). The ‘positive’ and ‘negative’ labels were displayed in the upper left and right corner of the computer screen. In the second block (32 trials), participants categorized words into the same attribute categories, but a second, target category of pictures was introduced in this block. Target labels were “monogamy” and “multiple partners”. These target category labels also appeared in the upper left and right corners of the screen, immediately below the attribute labels. The setup of the third (congruent, test) block (48 trials) was similar to the second block, except that the second block was presented as a practice block while the third block was presented as a test block. In the fourth block (32 trials), the positions of the target category labels (monogamy/multiple partners) on top of the screen were swapped. In order to categorize pictures, participants now had to press the opposite keys for the picture stimuli compared to the keys they had to use in blocks two and three. The positions of the “positive” and “negative” labels remained the same. The fifth block (48 trials) had the same setup as the fourth block of our IAT, but once again the fourth block was presented as a practice block while the fifth block was presented as a test block. The combinations of attributes under one key were counterbalanced, meaning one half of our sample first had “positive” and “monogamy” under one key at the second and third block, while the other half first had “positive” and “multiple partners” under one key in the second and third block. Table 1 provides a summary of the IAT procedure.

**Table 1:** Sequence of blocks for the IAT used in this study.

Block number	Block description	Type ofblock	‘z’ keycategory	‘m’ keycategory	Numberof trials
**1**	Single categorisation of target word	Practice	Positive	Negative	16
**2**	Combined categorisation	Practice	Positive and Monogamy	Negative and Multiple Partners	32
**3**	Combined categorisation	Test	Positive and Monogamy	Negative and Multiple Partners	48
**4**	Combined categorisation reversed	Practice reversed	Positive and Multiple Partners	Negative and Monogamy	32
**5**	Combined categorisation reversed	Test reversed	Positive and Multiple Partners	Negative and Monogamy	48

### Procedure/Protocol

Students were recruited via an email sent to the entire student body of Antwerp University. Students wanting to participate clicked on a link which took them to the study website which was hosted on the *Project Implicit*^®^ Web site (https://implicit.harvard.edu/). The first step on the study website was signing the informed consent. Participants then completed the C-IAT, and after this the explicit questionnaire. The IAT and explicit measures took approximately 15 minutes to complete.

**Explicit Questionnaire:** After completing the IAT the students were asked to complete a questionnaire pertaining to their sexual behavior and explicit attitudes to concurrency. These questions (variables they are intended to define) included: *How would you define your sexual orientation*? (Sexual orientation). Respondents had 4 responses available- Heterosexual, men who have sex with men (MSM), women who have sex with women (WSW) and other; *How many sex partners do you have*? (Current concurrency); *Were there any other times in your life when you had more than one sex partner at a time*? (Life time concurrency); *Do you think that any of your sex partners in the past 12 months have had other sex partners whilst they were in a sexual relationship with you*? (Partner concurrency); *Do you CURRENTLY have a ‘steady partner’*, *meaning you are not ‘single’*, *referring to partners that are not simply casual sex partners*? (Steady partner).

Endorsement of three statements investigating explicit attitudes towards concurrency was assessed using a scale from 1 (strongly disagree) to 5 (strongly agree): *It’s okay to have sex with others as long as your main partner does not find out* (Concealed concurrency); *If you are in a sexual relationship with someone*, *it’s okay to have sex with others as long as you are honest with your main partner about this* (Liberalist concurrency); *If my main partner has other sex partners*, *it is okay for me to have other partners as well* (Reactive concurrency).

### Statistical analysis

#### Individual level analyses

D600-scores of the IAT were calculated according to the standard protocol suggested by Greenwald et al. [49, 50]. Reaction times of the second target-attribute combination were subtracted from the first combination, correcting for combination sequence, and divided by the pooled standard deviation of all practice and test phases. Scores usually vary between -2 and +2, with high scores indicating strong implicit preferences for monogamy and concurrency, respectively, with zero indicating absence of preference, positive scores indicating a positive implicit association with concurrency (and a negative association with monogamy), and negative scores indicating a negative implicit association with concurrency (and a positive association with monogamy). Breakpoints for slight (0.15), moderate (0.35), and strong (0.65) preference for concurrency or monogamy were selected according to conventions for effect size [51]. Before calculating the D600 score, the minimum response time was set at 400 ms, the maximum response time at 2500 ms. Any responses below this interval were omitted while any responses above this interval were recoded to 2500 ms. Reaction times of incorrect answers were raised using a penalty of 600 ms.

We compared MSM with heterosexual men, WSW with heterosexual women. 54 individuals reported their sexual orientation to fall in the ‘other’ category. These likely reflect a heterogenous group and we therefore elected to conduct statistical comparisons between this group and other groups. The heterosexual men and women were chosen as the reference group as they were numerically the largest groups. We compared the distributions of C-IAT score (D600) between the various comparison groups using t-tests for independent samples.

In keeping with standard practice in this field, we used Cohen’s *d* as a measure of effect size. Pearson’s correlation was used to test the correlations between implicit and explicit attitudes as well as between these two and self-reported lifetime concurrency. Chi-square and t-tests were used to test differences between categorical and continuous variables.

**Multivariate analyses:** We used multivariate linear regression analyses to control for potential confounders in the relationship between C-IAT and sexual orientation/gender. In this model individuals were coded into one of six sexual orientation groups: heterosexual women, heterosexual men, WSW, MSM, ‘Other’ women and ‘Other’ men. The additional variables entered into these models were based on a literature review of potential confounders in the relationship between implicit associations and sexual orientation and/or gender. Potentially confounding demographic characteristics considered included current age (expressed as a continuous variable) [46, 52, 53], and ethnicity [31, 54]. Being in a committed, long-term relationship has been found to be negatively associated with number of sexual partners and concurrency [7, 55–60]. The number of sex partners in the past year [7, 61], the practice of concurrency or the belief that a partner had engaged in concurrency could all influence one’s implicit and explicit attitudes towards concurrency and have been shown to vary by sexual orientation and gender [6, 58, 60, 62–65].

The model was built by entering all the potential confounding factors described above simultaneously. 71 cases with missing data were dropped from the multivariate analysis.

**Correlates of concurrency:** The same model building process was used to examine how the correlates of concurrency varied by gender. The dependent variable was the C-IAT D600 score. Separate models were run for women and men.

#### Population level analyses

Pearson’s correlation was used to assess the population level correlations between the prevalence of current concurrency and implicit (mean D600) and explicit (mean values for each of the 3 variables considered separately) attitudes. The populations were defined according to self-reported sexual orientation and gender. The point-prevalence of concurrency at the time of the study was used in these analyses based on the recommendation by a number of authors that this be used as the most meaningful summary measure of concurrency prevalence [1, 36, 52, 58, 66].

A *p*-value of < 0.05 was considered statistically significant. All analyses were performed in Stata 13 (StataCorp LP, College Station, TX, USA). All procedures were approved by the institutional review boards of the Institute of Tropical Medicine (Antwerp) and the University of Antwerp (ITG-965/14).

## Results

### Participants’ characteristics

869 students completed the study. The mean age of participants was 22.95 years (Standard Deviation (SD) = 5.21) with no significant difference in age or ethnicity between heterosexual men, heterosexual women, WSW, MSM or Others—with the exception of WSW who were slightly younger than heterosexual women (Table 2). Whilst there was no difference between the heterosexual men and heterosexual women in terms of number of partners, concurrent partners and lifetime concurrent partners, heterosexual women were more likely to report that their partners had other partners (17.5% vs. 10.3%; *p* < 0.05). Samples sizes were small for the WSW (N = 20), MSM (N = 32) and ‘Other’ men (N = 13) and ‘Other’ women (N = 41). On the whole these groups reported more sex partners and higher rates of concurrency than their reference groups (all *p* values < 0.05 except for concurrency in MSM).

**Table 2:** Characteristics of study samples and implicit and explicit attitudes towards concurrency according to sexual orientation. No. (%) / Mean [Standard Deviation].

	Heterosexual Men	Heterosexual Women ^a^	WSW ^b^	MSM ^c^	Other Men	Other Women
***N***	268	464	20	32	10	41
**Gender (Women)**	0	464 (100)	20 (100)	0	0	41 (100)
**Age—mean [SD]**	23.6 [5.7]	22.6 [5.0]	21.7 [3.8] *	24.3 [6.0]	24.3 [5.9]	22.3 [3.1]
**Race/Ethnicity**						
**European/White**	265 (98.9)	449 (96.8)	19 (95)	32 (100)	10 (100)	40 (97.6)
**African/Black**	2 (0.8)	4 (0.9)	0	0	0	0
**Asian**	0	6 (1.3)	1 (5)	0	0	1 (2.4)
**Other**	1 (0.4)	5 (1.1)	0	0	0	0
**Sexual behavior**						
**No. partners last year—mean [SD]**	1.18 [1.48]	1.21 [1.49]	1.79 [1.36] *	5.52 [10.4] ***	2.50 [2.91]	1.48 [1.78]
**Steady partner (Yes)**	144 (54.6)	275 (59.8)	14 (70.0)	15 (46.9)	2 (20)	20 (48.8)
**Current concurrency**	5 (1.9)	9 (2.0)	3 (15) **	2 (6.3)	1 (10)	5 (12.5)
**Lifetime concurrency**	53 (20.1)	85 (18.5)	10 (50.0) **	19 (59.4) ***	2 (20)	17 (42.5)
**Partner concurrency**	27 (10.3)	80 (17.5) *	8 (42.1)	12 (37.5) *	2 (22.2)	10 (25)
**Implicit norms**						
**C-IAT**	-0.82 [0.39]	-0.82 [0.34]	-0.70 [0.44]***	-0.72 [0.43]***	-0.73 [0.35]	-0.34 [0.62]
**Cohen’s d** ^e^	-	0.016	0.144	0.160	NA	NA
**Explicit norms** ^d^						
**Concealed concurrency**	1.51 [0.77]	1.28 [0.56] ***	1.30 [0.57]	1.72 [0.99]	2.10 [1.19]	1.51 [0.64]
**Liberalist concurrency**	2.61 [1.24]	2.36 [1.22]	2.85 [1.31]	3.19 [1.20] *	3.00 [1.33]	3.59 [1.24]
**Reactive concurrency**	2.52 [1.24]	2.25 [1.17] *	2.35 [1.38]	2.84 [1.32]	3.5 [1.50]	3.05 [1.26]

*P<0.05,

** P<0.005

*** P<0.0005

^a^ P-values in Heterosexual Women column refer to comparisons with Heterosexual Men column

^b^ P-values in WSW (Women who have sex with women) column refer to comparisons with Heterosexual Women column

^c^ P-values in MSM (Men who have sex with men) column refer to comparisons with Heterosexual Men column

^d^ Explicit norms towards concurrency are reported as mean values of 5 scale measures with 5 indicating strongest approval of the specified attitude towards concurrency

^e^ Cohen’s d compares the difference in D600 effect size of heterosexual women with heterosexual men (2^nd^ column), WSW with heterosexual women (3^rd^ column), MSM with heterosexual men (4^th^ column), other men with heterosexual men (5^th^ column) and other women with heterosexual women (6^th^ column).

### Relationship between C-IAT and gender/sexual orientation

**Fig 1:**
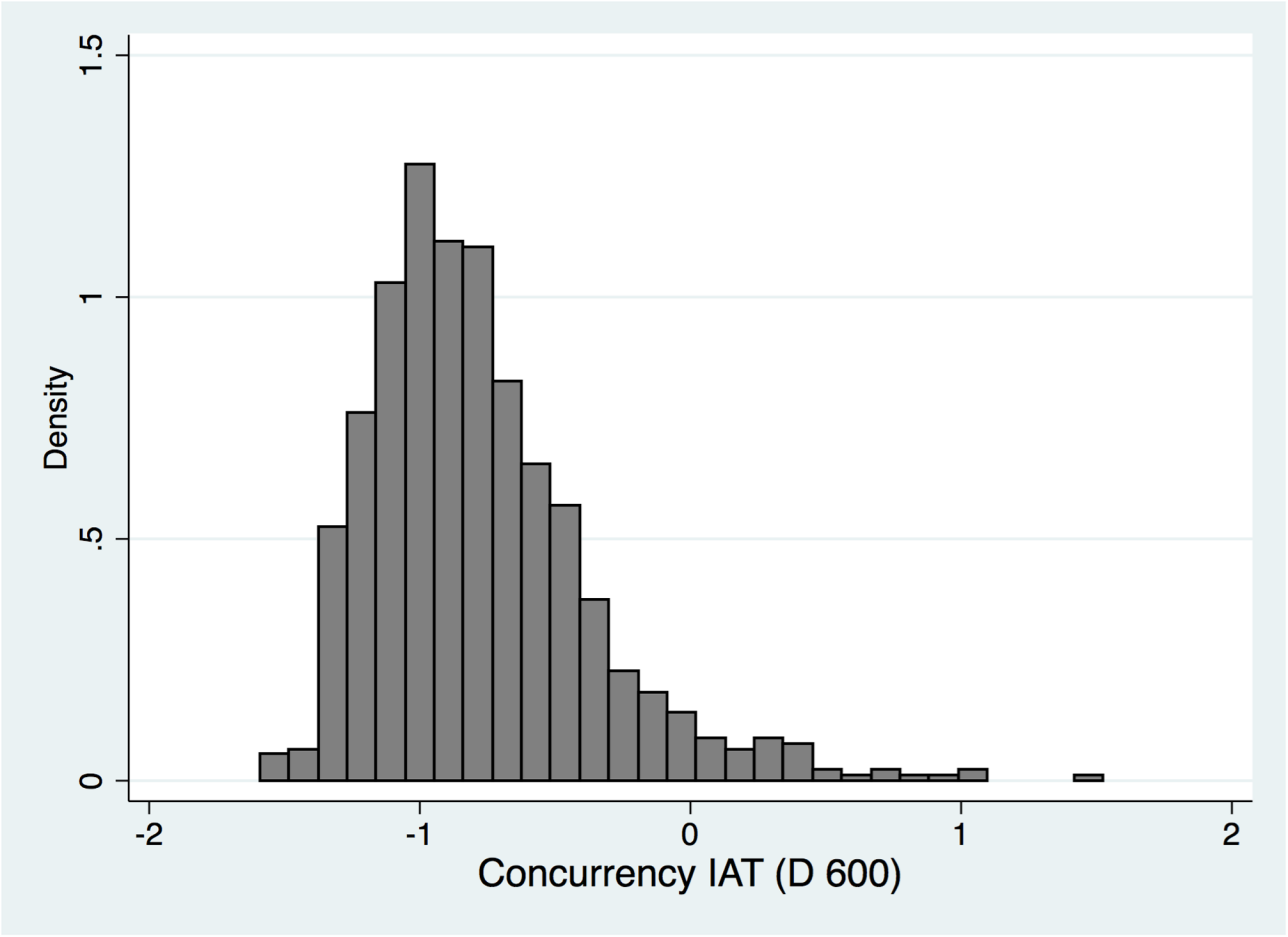
Distributions of concurrency implicit association test (D600) scores for 869 Belgian students.

The study participants C-IATs demonstrated a preference for monogamy (-0.78, SD = 0.41). 810 (93.2%) participants exhibited a preference for monogamy– 204 (23.5%) a slight and 606 (69.7%) a strong preference. C-IATs were pro-concurrency in 29 (4.2%) students. 30 (3.5%) participants had neutral C-IATs. The participants C-IAT scores were approximately normally distributed (Fig 1). There was no difference in C-IAT score between heterosexual men (-0.82, SD = 0.39) and heterosexual women (-0.82, SD = 0.34; Table 2 & Fig 1). WSW (-0.70, SD = 0.44) and MSM (-0.72, SD = 0.43) had stronger pro-concurrency D600-scores than their respective reference groups (all *p*-values < 0.0005). The effect size measure, Cohen’s *d*, varied between 0.02 (negligible effect size) to 0.16 (weak sized effect; Table 2).

Controlling for a number of potential confounders in multivariate analyses did not alter the key finding that there was no significant difference in the C-IAT between heterosexual women and men (*p* < 0.001; Table 3).

**Table 3:** Linear regression models predicting concurrency-implicit association test D-600 scores—Beta coefficients (95% confidence intervals).

	All individuals	Women	Men
***N***	798	501	295
**Age**	0.0011 (-0.0041–0.0063)	0.0017 (-0.0051–0.0085)	0.0010 (-0.0070–0.0090)
**Sexual Orientation**			
**Heterosexual women**	Ref	Ref	NA
**Heterosexual men**	-0.032 (-0.091–0.026)	NA	Ref
**WSW**	0.094 (-0.080–0.269)	-0.082 (-0.091–0.226)	NA
**MSM**	0.037 (-0.185–0.111)	NA	-0.023 (-0.177–0.131)
**Other men**	-0.030 (-0.269–0.208)	NA	-0.096 (-0.367–0.174)
**Other women**	0.441 (0.315–0.567)***	0.452 (0.326–0.579)***	NA
**Race/Ethnicity**			
**European/White**	Ref	Ref	Ref
**African/Black**	-0.207 (-0.507–0.093)	-0.153 (-0.518–0.212)	-0.365 (-0.884–0.155)
**Asian**	-0.201 (-0.481–0.080)	-0.181 (-0.461–0.096)	- ^#^
**Other**	0.132 (-0.198–0.462)	0.172 (-0.194–0.538)	0.064 (-0.691–0.812)
**Sexual behavior**			
**No. partners last year**	0.017 (0.005–0.029)**	0.006 (-0.026–0.039)	0.021 (0.008–0.346)**
**Steady partner (Yes)**	-0.024 (-0.079–0.031)	0.029 (-0.041–0.100)	-0.098 (-0.190–0.005)*
**Current concurrency**	0.064 (-0.099–0.226)	0.167 (-0.036–0.370)	-0.095 (-0.383–0.193)
**Partner concurrency**	-0.028 (-0.109–0.052)	0.013 (-0.090–0.115)	-0.095 (-0.245–0.055)
**Explicit norms**			
**Concealed concurrency**	0.061 (0.103–0.181)**	0.041 (-0.021–0.102)	0.110 (0.048–0.172)**
**Liberalist concurrency**	0.016 (0.009–0.042)	-0.011 (-0.044–0.021)	0.046 (0.003–0.088)*
**Reactive concurrency**	0.024 (-0.002–0.052)	0.051 (0.017–0.085)**	0.129 (-0.558–0.029)

*P<0.05,

** P<0.005

*** P<0.0005;

^#^ Omitted due to collinearity

Heterosexual men were more likely than heterosexual women to express explicit attitudes tolerant of concealed concurrency and reactive concurrency (Table 2). MSM were more likely to be tolerant of liberalist concurrency.

### Relationship between implicit and explicit attitudes and reported concurrency

#### Implicit concurrency

**Individual level:** In 5 of 6 comparisons, the C-IAT score was weakly positively correlated with reporting current concurrency (Table 4). Only in the case of ‘Other’ women was this association statistically significant.

**Table 4:** Pearson’s correlations between implicit and explicit attitudes towards concurrency according to sexual orientation.

	Heterosexual Men	Heterosexual Women ^a^	WSW ^b^	MSM ^c^	Other Men	Other Women
***N***	268	464	20	32	10	41
**Correlation: Lifetime concurrency vs C-IAT**	0.14 *	0.03	0.14	0.07	-0.61	0.28
**Correlation: Current concurrency vs C-IAT**	0.04	0.05	0.16	0.12	-0.24	0.44**
**Correlation: Lifetime concurrency vs explicit attitudes**						
**Concealed concurrency**	0.36 ***	0.26 ***	0.36	0.28	0.84**	0.36*
**Liberalist concurrency**	0.14 *	0.20 ***	0.27	-0.08	0.00	0.20
**Reactive concurrency**	0.11	0.16 ***	0.41 *	0.10	0.52	0.15
**Correlation: Current concurrency vs explicit attitudes**						
**Concealed concurrency**	0.26***	0.15**	0.02	0.21	0.84	-0.17
**Liberalist concurrency**	0.01	0.03	0.20	-0.07	0.34	0.21
**Reactive concurrency**	0.00	0.11*	0.27	-0.04	0.21	0.37*
**Correlation: C-IAT vs explicit attitudes**						
**Concealed concurrency**	0.25 ***	0.15 **	0.15	0.39 *	0.37	0.14
**Liberalist concurrency**	0.19 **	0.09 *	-0.01	0.23	0.36	0.28
**Reactive concurrency**	0.13 *	0.19 ***	0.44 *	0.18	0.01	0.08

*P<0.05,

** P<0.005

*** P<0.0005

^a^ P-values in Heterosexual Women column refer to comparisons with Heterosexual Men column

^b^ P-values in WSW (Women who have sex with women) column refer to comparisons with Heterosexual Women column

^c^ P-values in MSM (Men who have sex with men) column refer to comparisons with Heterosexual Men column

**Population level:** The prevalence of current concurrency was non-significantly positively correlated with the mean C-IAT by sexual orientation category (r = 0.63, *p* = 0.18). A stronger implicit pro-monogamy attitude was associated with a lower reported prevalence of concurrent behavior. The removal of the outlier group (‘Other’ women) strengthened this correlation (r = 0.89, *p* = 0.04).

#### Explicit concurrency

Correlations between current concurrency and explicit measures were generally stronger than those using implicit attitudes (r = 0.11 to 0.36 for heterosexual men and women).

### Relationship between implicit and explicit measures

The C-IAT was positively correlated with all three explicit measures of concurrency. In the heterosexual men and women groups the strength of this correlation was 0.09 to 0.25 (all *p* < 0.05).

### Correlates of C-IAT

Amongst men, a pro concurrency C-IAT score was associated with reporting more partners in the past year (*p* = 0.002), not having a steady partner (*p* = 0.040) and expressing favorable attitudes towards concealed- and liberal-concurrency (*p* = 0.001 and *p* = 0.029, respectively).

In women, a pro concurrency C-IAT score was positively associated with being in the ‘Other’ sexual orientation category (*p* < 0.001) and reporting more sympathetic views to reactive concurrency (*p* = 0.004).

## Discussion

The prevalence of concurrency has been found to be an important determinant of the prevalence of a wide number of STIs [4, 5, 67]. Qualitative and quantitative studies have reached different conclusions as whether or not higher concurrency rates are underpinned by explicit attitudes receptive to concurrency [7, 27, 28, 32, 68]. Concurrency is a sensitive domain and therefore particularly sensitive to a social desirability bias [66]. Because measures of implicit cognition assess cognitive processes unavailable to introspection less affected by these problems [40]. Implicit measures may therefore be better predictors of behavior than explicit measures in these sensitive domains [40, 43]. These considerations provide the rationale for assessing implicit associations to concurrency. In this initial test of the C-IAT in a large sample of young Belgian students we found no difference between men and women but moderately sized differences between sexual orientations in implicit attitudes to concurrency. This relatively low concurrency prevalence population (compared to similar aged populations in high HIV prevalence populations [46, 47]) exhibited a strong implicit preference for monogamy.

### Relationship between C-IAT and gender/sexual orientation

The lack of difference in C-IAT results between men and women provides evidence that there are few or no differences in implicit attitudes towards concurrency by gender in this population of university students. We did however find differing associations between C-IAT and explicit attitudes by gender. No overlap was found between genders in the factors found to be significantly correlated with the C-IAT. In men number of partners, absence of a steady partner and expressing favorable attitudes towards concealed- and liberal-concurrency whilst in women a favorable attitude to reactive concurrency were associated with pro-concurrency C-IATs. These may point to differences in how men and women process cognitions pertaining to non-monogamous sex/relationships [69]. A number of studies have found that for both men and women the perception or knowledge that ones partner has engaged in concurrency is a strongly associated (adjusted odds ratios of up to 8) with respondent concurrency [7, 10, 11, 46, 58, 70]. At least four mechanisms for this association have been proposed [58, 70]. A number of studies have found evidence that reactive concurrency is particularly important [46, 58, 70]. Although we cannot ascertain the direction of causation, our results suggest the possibility that the psychological mechanisms underpinning the association between partner and respondent concurrency vary by gender.

Although there were statistically significant differences between heterosexual men and MSM and between heterosexual women and WSW, these were of a small magnitude. The C-IATS of all the groups were pro-monogamy.

### Is the C-IAT a useful predictor of concurrency?

At an individual level only a weak association was found between C-IAT and concurrent behavior. At a population level there was possible correlation between self-reported concurrency and implicit attitudes towards concurrency. A particularly striking finding of this study was that for both men and women the population distribution of C-IATs followed a normal distribution with 92.3% of the sample revealing an implicit preference for monogamy. This is perhaps not a surprising finding given that until recent times any form of non-monogamy was proscribed and stigmatized in Western Europe [71–73]. It would therefore be instructive to reproduce this study in populations in regions such as sub-Saharan Africa, which have histories of greater tolerance to various forms of concurrent partnering [27, 53, 68]. If these populations were found to have similar C-IATs to the Belgian students then implicit attitudes to concurrent partnering may be universally pro-monogamy. If, however, these populations have C-IATs that are more pro-concurrency than the Belgians, then this would open up important new lines of enquiry such as what the correlates and relevance of these differences are and at what age these differences emerge. Insights gained from such studies may be of utility to the design of campaigns addressing high concurrency rates [74, 75]. Monitoring C-IATs may also be of utility to assess the efficacy of these campaigns [74].

### Correlation between C-IAT and explicit attitudes

The C-IAT was weakly positively correlated with all 3 explicit measures of concurrency. The correlations found were not dissimilar to those reported from a meta-analysis that included 126 independent correlations [76]. The correlations between implicit and explicit attitudes in this meta-analysis ranged from r = –.25 to r = .60, with a mean correlation of .19 [76]. The correlations we found between explicit attitudes to concurrency and reported concurrency were similar to those found in other studies that have assessed this [32, 77].

### Limitations

There are a number of limitations to this study, including the fact that all the participants were young and from a single university in Belgium, thus limiting the generalizability of the findings. The sample sizes for all groups except the heterosexual men and women were small. The ‘other’ men and women groups are likely to be constituted by a number of different sexualities, such as bisexuals and transsexuals, and hence the results pertaining to these groups should be treated with caution. All students could participate in the study including those who were not sexually experienced. This could have influenced a number of variables including the prevalence of concurrency and implicit and explicit attitudes towards concurrency. The C-IAT may have had a heterosexual bias in the sense that whilst the words and cartoons used were gender-neutral the photographs used could be conceived of as representing heterosexual relations only. This may have confounded the association we found between sexual orientation and C-IAT. Studies have found that respondents may not be accurate in how self-reports of their concurrency and that of their partners [38, 78]. These could introduce misclassification biases in our analyses. Being a cross-sectional study, no causal inferences can be made based on any associations found.

### Conclusion

We found no evidence for variations in implicit attitudes towards concurrency by gender but moderate differences between different sexualities. There was a possible ecological-level correlation between self-reported concurrency and implicit attitudes towards concurrency. Further research should prioritize assessing what implicit attitudes to concurrency are in a more diverse range of populations including those with a history of greater tolerance of forms of non-monogamy.

## Supporting information

S1 IAT Test (English) concurrency implicit association test. (GZ)

S2 IAT Test (Dutch) concurrency implicit association test. (GZ)

S1 Data set Concurrency IAT test results. (DTA)

S1 File Box A and Box B. (DOCX)
