# Supplementary figures and images for: Implicit attitudes to sexual partner concurrency vary by sexual orientation but not by gender—A cross sectional study of Belgian students
Source: PLoS One. 2018 May 8;13(5):e0196821. doi: 10.1371/journal.pone.0196821 (PMC5940213; doi:10.1371/journal.pone.0196821)

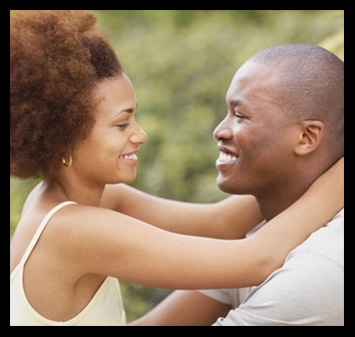

Supplement: S1 IAT Test — (GZ) [file pone.0196821.s001.tar.gz › pool/stim1.jpg]

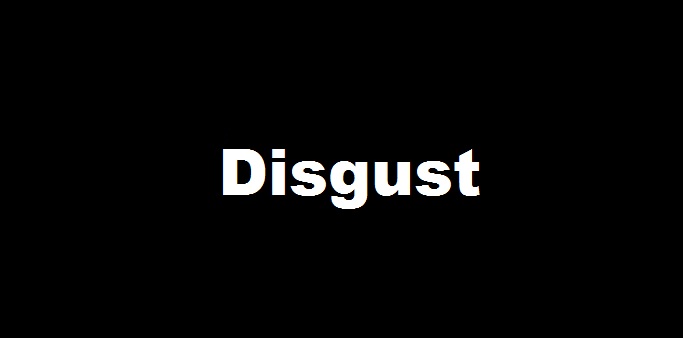

Supplement: S1 IAT Test — (GZ) [file pone.0196821.s001.tar.gz › pool/stim10.jpg]

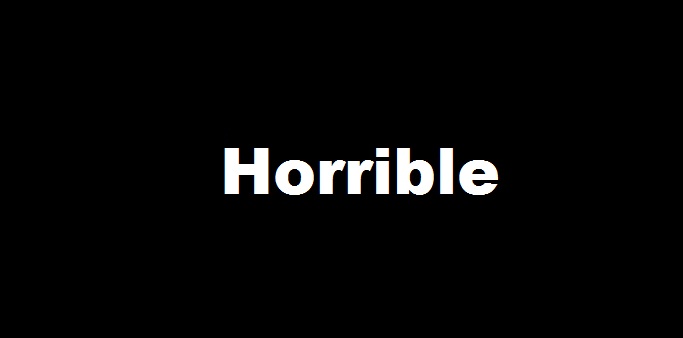

Supplement: S1 IAT Test — (GZ) [file pone.0196821.s001.tar.gz › pool/stim11.jpg]

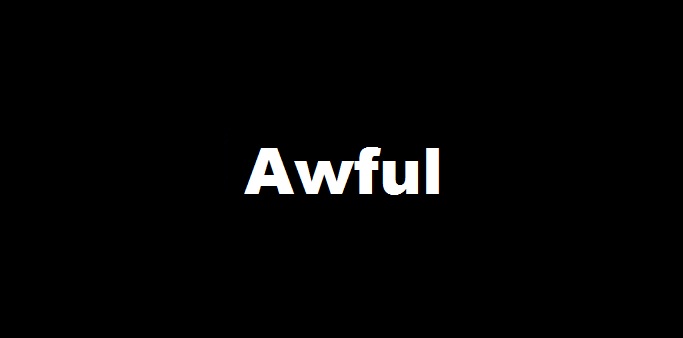

Supplement: S1 IAT Test — (GZ) [file pone.0196821.s001.tar.gz › pool/stim12.jpg]

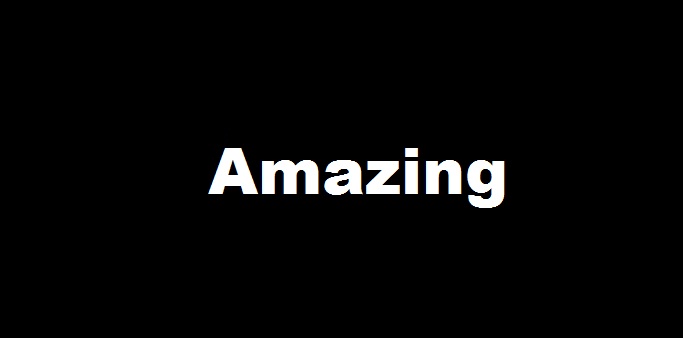

Supplement: S1 IAT Test — (GZ) [file pone.0196821.s001.tar.gz › pool/stim13.jpg]

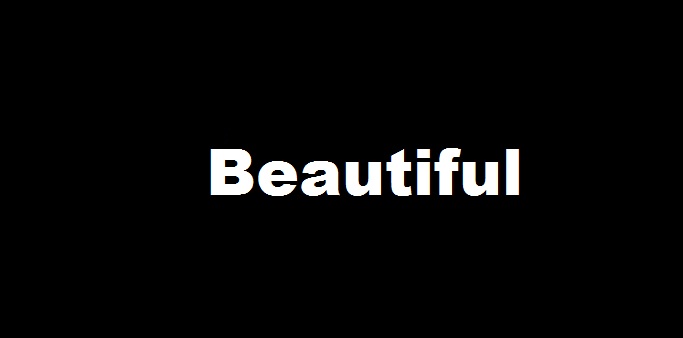

Supplement: S1 IAT Test — (GZ) [file pone.0196821.s001.tar.gz › pool/stim14.jpg]

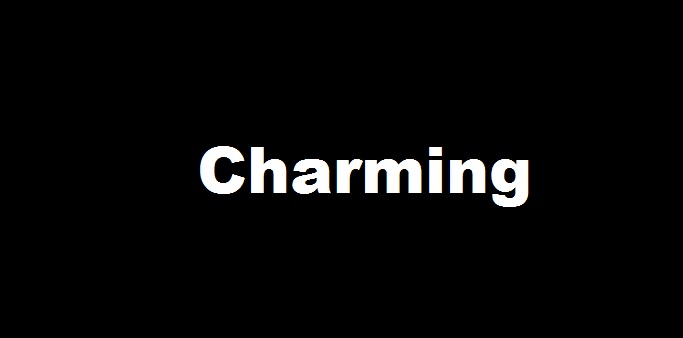

Supplement: S1 IAT Test — (GZ) [file pone.0196821.s001.tar.gz › pool/stim15.jpg]

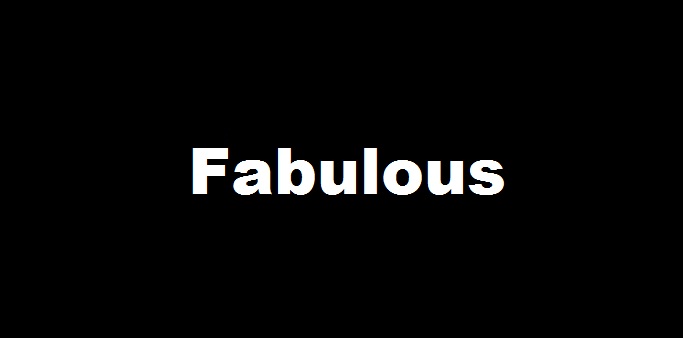

Supplement: S1 IAT Test — (GZ) [file pone.0196821.s001.tar.gz › pool/stim16.jpg]

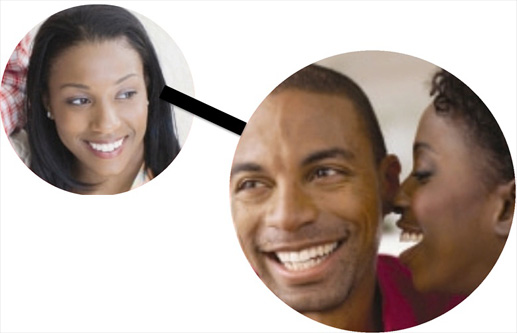

Supplement: S1 IAT Test — (GZ) [file pone.0196821.s001.tar.gz › pool/stim2.jpg]

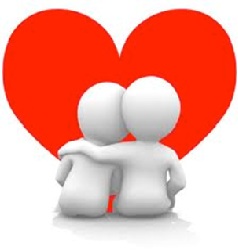

Supplement: S1 IAT Test — (GZ) [file pone.0196821.s001.tar.gz › pool/stim3.jpg]

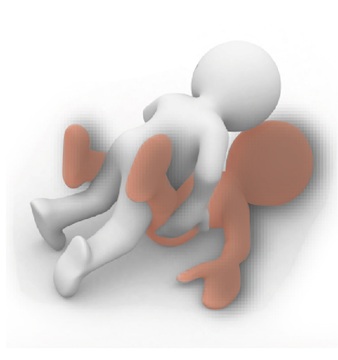

Supplement: S1 IAT Test — (GZ) [file pone.0196821.s001.tar.gz › pool/stim4.jpg]

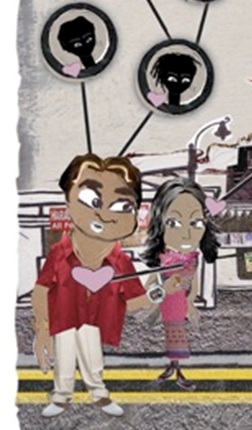

Supplement: S1 IAT Test — (GZ) [file pone.0196821.s001.tar.gz › pool/stim5.jpg]

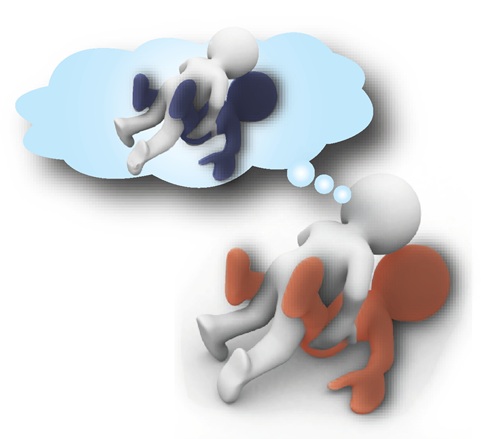

Supplement: S1 IAT Test — (GZ) [file pone.0196821.s001.tar.gz › pool/stim6.jpg]

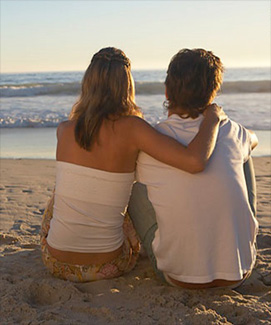

Supplement: S1 IAT Test — (GZ) [file pone.0196821.s001.tar.gz › pool/stim7.jpg]

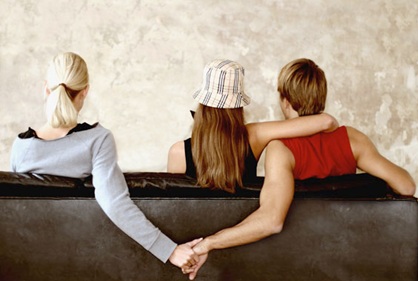

Supplement: S1 IAT Test — (GZ) [file pone.0196821.s001.tar.gz › pool/stim8.jpg]

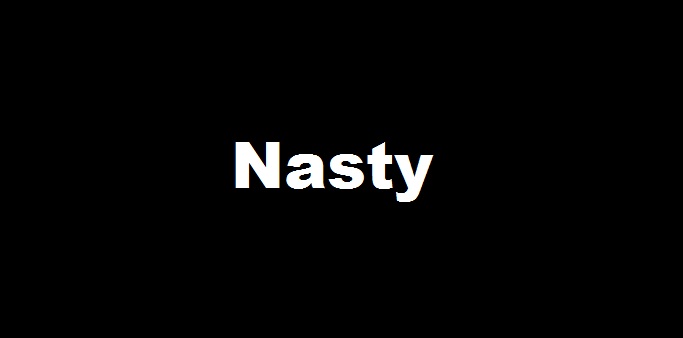

Supplement: S1 IAT Test — (GZ) [file pone.0196821.s001.tar.gz › pool/stim9.jpg]

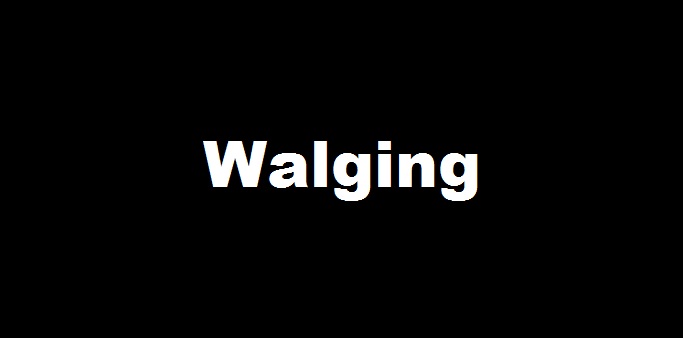

Supplement: S2 IAT Test — (GZ) [file pone.0196821.s002.tar.gz › pool/stim10.jpg]

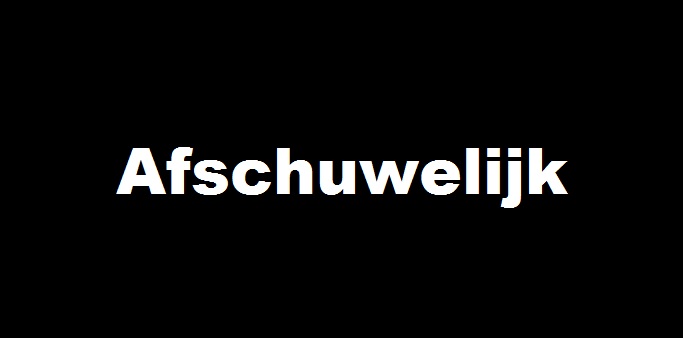

Supplement: S2 IAT Test — (GZ) [file pone.0196821.s002.tar.gz › pool/stim11.jpg]

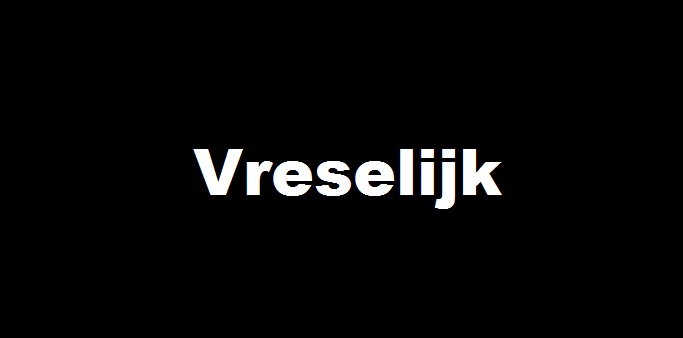

Supplement: S2 IAT Test — (GZ) [file pone.0196821.s002.tar.gz › pool/stim12.jpg]

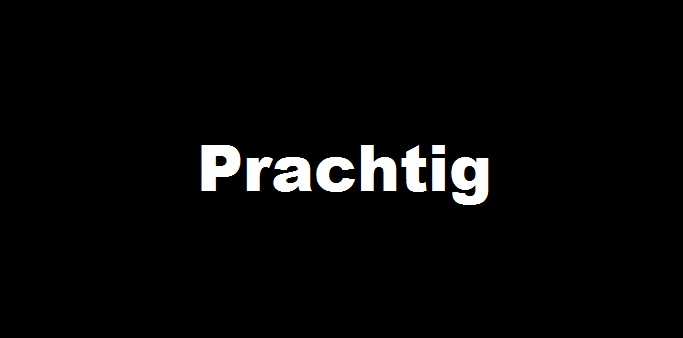

Supplement: S2 IAT Test — (GZ) [file pone.0196821.s002.tar.gz › pool/stim13.jpg]

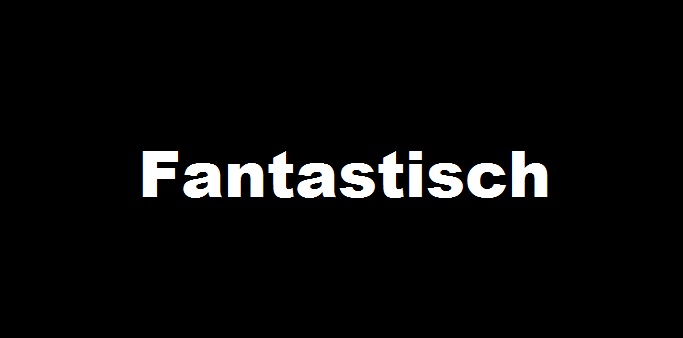

Supplement: S2 IAT Test — (GZ) [file pone.0196821.s002.tar.gz › pool/stim14.jpg]

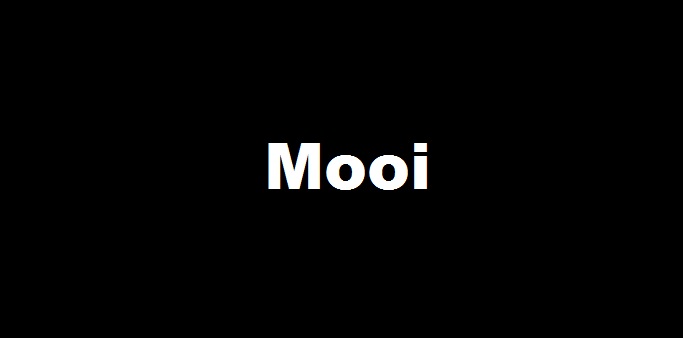

Supplement: S2 IAT Test — (GZ) [file pone.0196821.s002.tar.gz › pool/stim15.jpg]

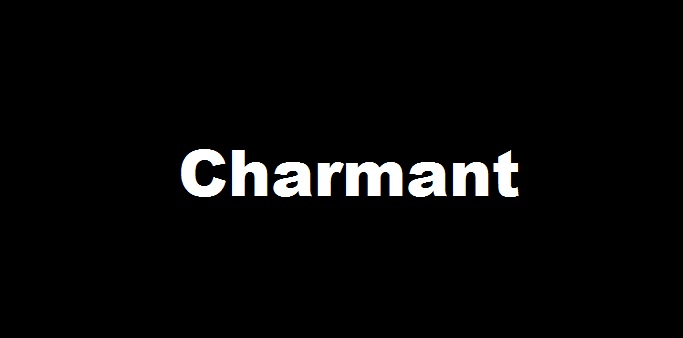

Supplement: S2 IAT Test — (GZ) [file pone.0196821.s002.tar.gz › pool/stim16.jpg]

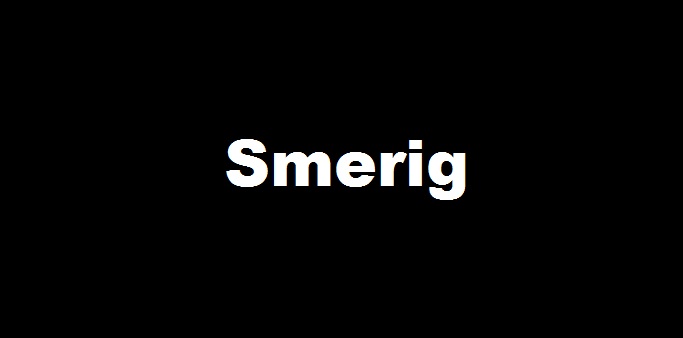

Supplement: S2 IAT Test — (GZ) [file pone.0196821.s002.tar.gz › pool/stim9.jpg]
