# Supplementary material for: Implicit attitudes to sexual partner concurrency vary by sexual orientation but not by gender—A cross sectional study of Belgian students
Source: PLoS One. 2018 May 8;13(5):e0196821. doi: 10.1371/journal.pone.0196821 (PMC5940213; doi:10.1371/journal.pone.0196821)
Supplement: S1 File — (DOCX) [file pone.0196821.s004.docx]

**S1 File**

**Table of Contents**

**Box A. Stimuli used in Concurrency IAT Test in English (a) and Dutch (b) Pg 2**

**Box B: Instructions for running Concurrency – Implicit Association Test Pg 8**

**Box A. Stimuli used in Concurrency IAT Test in English (a) and Dutch (b) Pg 2**

a)

b)

**Box B: Instructions for running Concurrency – Implicit Association Test**

1. Download and install Open Sesame Software V2.9.7 or later from:

<http://osdoc.cogsci.nl/2.9/getting-opensesame/download/>

1. Open and run “Concurrency IAT file” from within Open Sesame
